# Astrocyte-Specific Genes Are Generally Demethylated in Neural Precursor Cells Prior to Astrocytic Differentiation

**DOI:** 10.1371/journal.pone.0003189

**Published:** 2008-09-11

**Authors:** Izuho Hatada, Masakazu Namihira, Sumiyo Morita, Mika Kimura, Takuro Horii, Kinichi Nakashima

**Affiliations:** 1 Laboratory of Genome Science, Biosignal Genome Resource Center, Institute for Molecular and Cellular Regulation, Gunma University, Maebashi, Japan; 2 Laboratory of Molecular Neuroscience, Graduate School of Biological Sciences, Nara Institute of Science and Technology, Ikoma, Japan; The Babraham Institute, United Kingdom

## Abstract

Epigenetic changes are thought to lead to alterations in the property of cells, such as differentiation potential. Neural precursor cells (NPCs) differentiate only into neurons in the midgestational brain, yet they become able to generate astrocytes in the late stage of development. This differentiation-potential switch could be explained by epigenetic changes, since the promoters of astrocyte-specific marker genes, glial fibrillary acidic protein (*Gfap*) and *S100β*, have been shown to become demethylated in late-stage NPCs prior to the onset of astrocyte differentiation; however, whether demethylation occurs generally in other astrocyctic genes remains unknown. Here we analyzed DNA methylation changes in mouse NPCs between the mid-(E11.5) and late (E14.5) stage of development by a genome-wide DNA methylation profiling method using microarrays and found that many astrocytic genes are demethylated in late-stage NPCs, enabling the cell to become competent to express these genes. Although these genes are already demethylated in late-stage NPCs, they are not expressed until cells differentiate into astrocytes. Thus, late-stage NPCs have epigenetic potential which can be realized in their expression after astrocyte differentiation.

## Introduction

DNA methylation usually occurs in mammalian cells at CpG dinucleotides and approximately 60–90% of cytosines at these sites are methylated [1]. Most CpG-rich DNA fragments, or CpG islands in genes, have been thought to remain unmethylated even in cell types that do not express the genes [2]. However, changes in DNA methylation have been sporadically observed in CpG islands during development and are thought to play important roles in the regulation of cell type-specific gene expression [3], [4]. DNA methylation also participates in the regulation of differentiation and embryonic development [5]. For example, inactivation of *Oct3/4* (*Pou5f1*) and *Nanog* genes by DNA methylation is important for early development [6], [7]. These genes are essential for maintaining pluripotency of embryonic stem (ES) cells and early embryos [8], [9], [10] and are also known as two of four genes which have been shown to reprogram somatic cells to pluripotent stem (iPS) cells with the essential characteristics of embryonic stem (ES) cells [11].

Epigenetic modification is also thought to play an important role in altering the differentiation potential of neural precursor cells (NPCs). NPCs differentiate only into neurons in the midgestational brain, while they become able to generate astrocytes as gestation proceeds [12]. The Janus kinase (JAK) signal transducer and activator of the transcription (STAT) pathway, which is activated by cytokines, including leukemia inhibitory factor (LIF), can effectively induce astrocyte differentiation [13], [14]. A particular cytosine residue within a STAT3-binding site in the astrocyte-specific marker glial fibrillary acidic protein (*Gfap*) gene promoter is highly methylated in NPCs midgestation (E11.5) when astrogenesis does not normally occur, while it becomes demethylated in late-stage (E14.5) NPCs that are prone to differentiating into astrocytes [15]. Although these late-stage (E14.5) NPCs with demethylated promoter do not express *Gfap*, the expression can be upregulated upon differentiation by leukemia inhibitory factor (LIF), which induces STAT3-activating cytokines [13], [14].

Previously, we developed a genome-wide DNA methylation analysis called Microarray-based Integrated Analysis of Methylation by Isoschizomers (MIAMI) using a microarray with genome-wide probes and used for several applications [16], [17]. With this method, we detected DNA methylation using the methylation-sensitive restriction enzyme *Hpa* II and its methylation-insensitive isoschizomer *Msp* I.

Although the demethylation of *Gfap* is known in late-stage NPCs prior to astrocyte differentiation [15], it is not known whether other genes confer this astrocytic property to cells. To study the role of DNA methylation in altering the differentiation potential of NPCs, here we analyzed DNA methylation changes in mouse NPCs between the mid-(E11.5) and late (E14.5) stages of development by a genome-wide DNA methylation profiling method using microarrays. We also compared the methylation status with that of postnatal day 1 (P1) astrocytes.

## Results and Discussion

### Genome-wide profiling of DNA methylation

**Figure 1:**
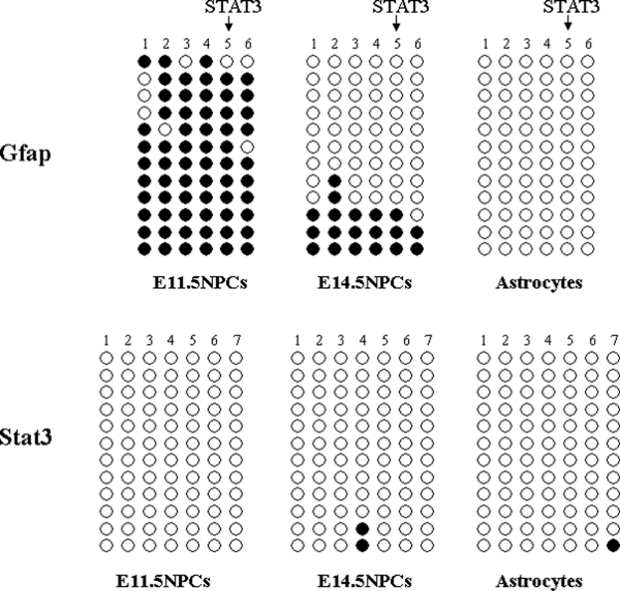
DNA methylation of *Gfap* and *Stat3* gene. DNA methylation was analyzed by bisulfite sequencing. Closed circles indicate methylated CpG sites and open circles indicate unmethylated CpG sites. STAT3 binding sites are indicated by arrows.

**Figure 2:**
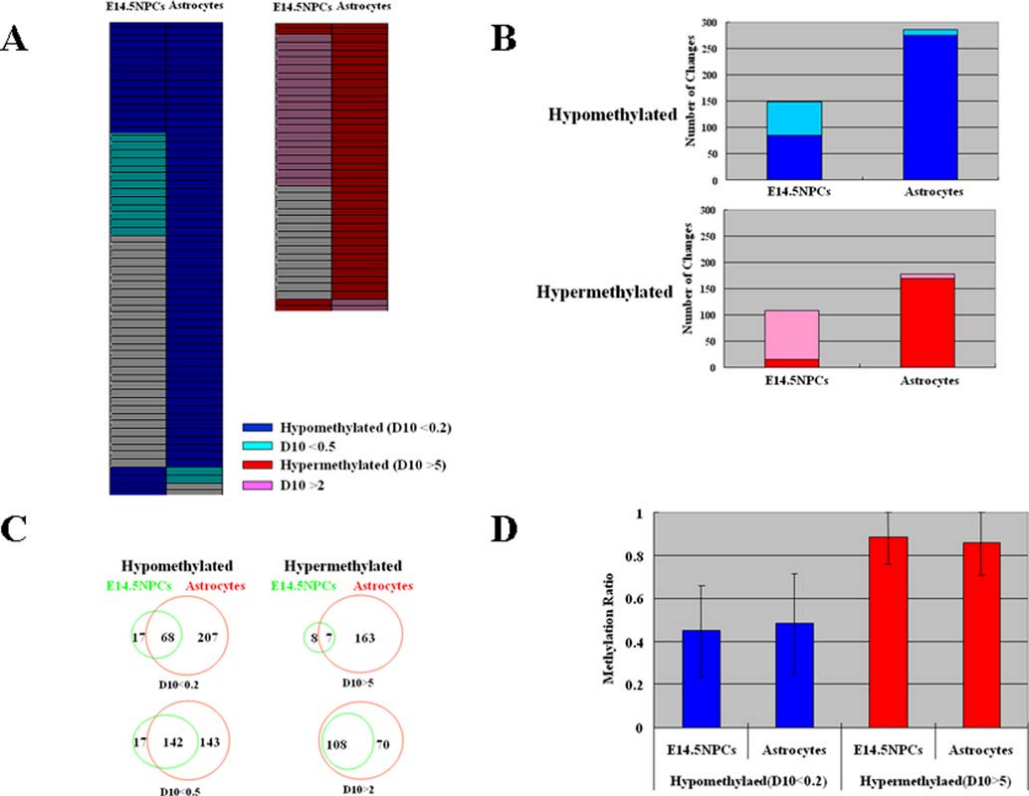
Genome-wide profiling of DNA methylation. (A) Hypomethylated and hypermethylated genes in E14.5 NPCs and astrocytes compared to E11.5 NPCs. Each row indicates each probe and each column indicates E14.5 NPCs or astrocytes. Probes with methylation changes are indicated in color. D10 is a value indicating the difference between methylation-sensitive *Hpa* II cleavage and methylation-insensitive *Msp* I cleavage. (B) Number of methylation changes in E14.5 NPCs and astrocytes compared to E11.5 NPCs. Numbers of methylation changes indicated in Fig. 1A were counted and indicated by bars. (C) Venn diagram of the number of probes with methylation changes in E14.5 NPCs and astrocytes compared to E11.5 NPCs. (D) Absolute levels of DNA methylation analyzed by MIAMI presented as methylation ratio. Methylation ratios of hypermethylated and hypomethylated probes are presented as average±standard deviation.

**Figure 3:**
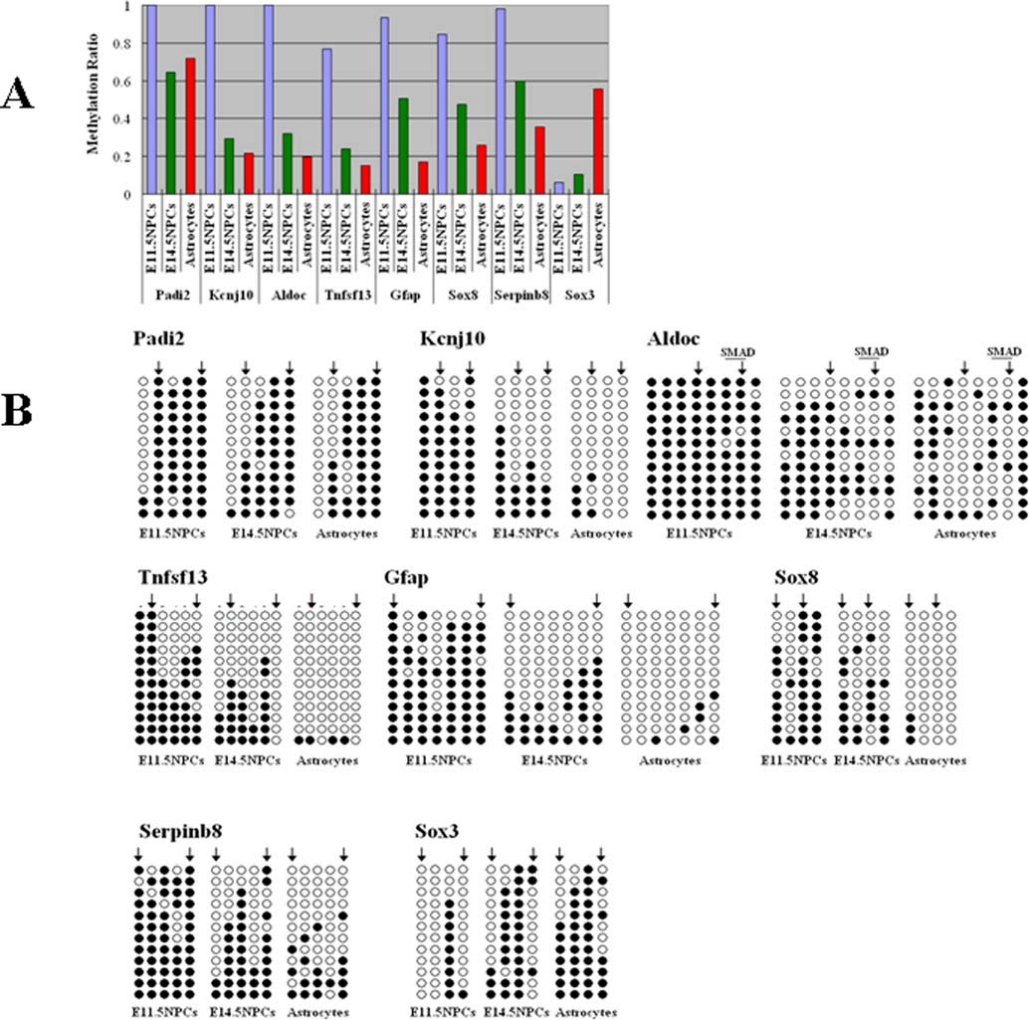
Confirmation of genome-wide methylation profiling by MIAMI. (A) Methylation ratios of seven hypomethylated and one hypermethylated gene compared to E11.5 NPCs. Methylation ratios are calculated by MIAMI data. (B) Confirmation of seven hypomethylated genes and one hypermethylated gene by bisulfite sequencing analysis. Closed circles indicate methylated CpG sites and open circles indicate unmethylated CpG sites. Positions of *Hpa* II sites are indicated as arrows.

Methylation changes in the differentiation potential switch of NPCs were analyzed by comparing mid-(E11.5) and late (E14.5)-stage NPCs using the MIAMI method [16]. We also compared the methylation status with that of postnatal day 1 (P1) astrocytes. Before analysis, the key genes involved in astrocyte differentiation, such as *Gfap* and *Stat3*, were analyzed for DNA methylation in the sample we used (Fig. 1). As described previously [15], the STAT3-binding site of *Gfap* gene was demethylated in E14.5 NPCs prior to astrocyte differentiation. On the other hand, the promoter of *Stat3* gene was unmethylated throughout differentiation. The microarray used consisted of probes chosen from the Agilent promoter array using an eArray system (http://earray.chem.agilent.com/earray/). The probes are located on *Hpa* II fragments of less than 1 kilobasepair (kb) and cover 14,543 genes. Probes which showed methylation changes at least in E14.5 NPCs or astrocytes compared to E11.5 NPCs are presented in Fig. 2A and Table S1 (Name of the probes and genes were indicated in Table S1). As shown in Fig. 2B, E14.5 NPCs are hypomethylated in 85 probes (80 genes) and hypermethylated in 15 probes (15 genes). On the other hand, astrocytes are hypomethylated in 275 probes (256 genes) and hypermethylated in 170 probes (152 genes). The reliability of the analysis was confirmed by bisulfite sequencing analysis of eight genes (Fig. 3). The methylation ratio analyzed by MIAMI (Fig. 3A) had good correlation with the methylation of two *Hpa* II sites adjacent to the probes (Fig. 3B). Interestingly 80% of the probes hypomethylated in E14.5 NPCs are also hypomethylated in astrocytes. (Fig. 2C). If we extend the hypomethylation change criteria to the threshold level (D10<0.5, usually we use D10<0.2 for hypomethylation), 89% of the probes hypomethylated in E14.5 NPCs are also hypomethylated in astrocytes. In other words, 48% of the hypomethylated probes in astrocytes are also hypomethylated in E14.5 NPCs. These include a probe for an astrocyte-specific marker gene, *Gfap*, which was previously shown to be hypomethylated both in E14.5 NPCs and astrocytes [15]. However, another hypomethylated astrocyte marker, S100β [18], was not detected by the MIAMI method because the DNA sequence with methylation change does not contain any *Hpa* II sites, leading to underestimation of the total number of genes actually demethylated in E14.5 NPCs. Nevertheless, including the genes undetectable by MIAMI, many hypomethylated sequences in astrocytes are already demethylated in E14.5 NPCs, which are competent to differentiate into astrocytes. These include important genes for astrocyte-specific function or phenotype in addition to an astrocyte-specific marker gene, *Gfap*. For example, Aldolase C encodes a member of the class I fructose-biphosphate aldolase gene family specific to astrocytes (*Aldoc*, 19). The demethylated region of *Aldoc* is located on exon 1. We further confirmed this result by bisulfite sequencing and found that *Aldoc* was methylated in E11.5 NPCs and demethylated in E14.5 NPCs and astrocytes (Fig. 3B). Another example, *Kcnj10* (*Kir4.1*), is widely expressed in astrocytes throughout the brain [20]. The product of this gene is absent in immature proliferating cells, and progressive expression of the genes correlates with astrocyte differentiation, which is characterized by the establishment of a negative membrane potential and exit from the cell cycle. *Kcnj10* (*Kir4.1*) encodes a member of the inward rectifier-type potassium channel family, characterized by having a greater tendency to allow potassium to flow into, rather than out of, a cell, resulting in negative membrane potential. The encoded protein is responsible for potassium buffering action, which is a major function of astrocytes and is also responsible for promoting differentiation and inhibiting cell growth [20], [21]. Mutations in this gene have been associated with seizure susceptibility of common idiopathic generalized epilepsy syndromes [22], [23]. *Sparcl1* is known as an astrocyte marker colocalized with *Gfap* [24] and is also known as a candidate gene for multiple sclerosis [25]. *Cbs* and *BC055107* are also known as astrocyte markers [26], [27]. Thus, we found that demethylation occurred not only in *Gfap* but also in the other genes involved in astrogenesis prior to astrocyte differentiation.

When comparing methylation status between E14.5 NPCs and astrocytes, it is important to know what percentage of the E14.5 NPCs actually gives rise to astrocytes. After 4 day-culture in the presence of LIF, 30–40% of E14.5 NPCs become GFAP-positive, yet this does not necessary mean that *Gfap* promoter is methylated in all GFP-negative cells. We have previously shown that STAT site within the *Gfap* promoter was virtually unmethylated in E14.5 NPCs during 4-day culture in the proliferating condition. They, however, could not give rise to 100% of GFAP-positive cells [15]. These findings suggest the existence of other factors that inhibit astrocyte differentiation. Thus, it is conceivable that demethylation is necessary but not sufficient for NPCs to differentiate into GFAP-positive astrocytes.

Absolute levels of DNA methylation were analyzed by MIAMI and were presented as average methylation ratio for hypomethylated and hypermethylated genes compared to E11.5 NPCs (Fig. 2D). On average, hypermethylated genes were nearly fully methylated as expected. However, hypomethylated genes had some methylation on average. In addition, some hypomethylated genes, specially, *Aldoc* had highly mosaic pattern, suggesting a possibility that equilibrium state may have been reached rather than DNA demethylation (Fig. 3B).

A hallmark for Polycomb-mediated repression is methylation of lysine 27 histone H3 (H3K27), which is set up by the Polycomb repressive complex2 [28], [29]. Many polycomb targets in embryonic stem cells (ESCs) have been shown to reside in a chromatin state characterized not only by the dual presence of repressive H3K27 methylation but also by active H3K4 methylation [30]. In neural differentiation, the promoters marked by H3K27 methylation in ESCs are more likely to become de novo DNA methylated [31]. Therefore, we analyzed the histone methylation status in ESCs for our hypermethylated genes in astrocytes compared to E11.5 NPCs using the published ESCs data [32]. We found that promoter that were marked by H3K27 in ESCs were significantly enriched in hypermethylated genes in astrocytes (P<1×10^−14^).

### Expression changes in NPCs and astrocytes

**Figure 4:**
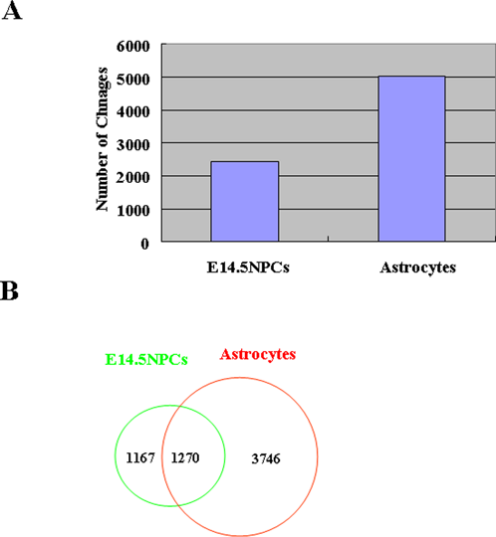
Expression analysis by microarray. (A) Number of upregulated genes in E14.5 NPCs and astrocytes compared to E11.5 NPCs. (B) Venn diagram of the number of upregulated genes in E14.5 NPCs and astrocytes compared to E11.5 NPCs.

**Figure 5:**
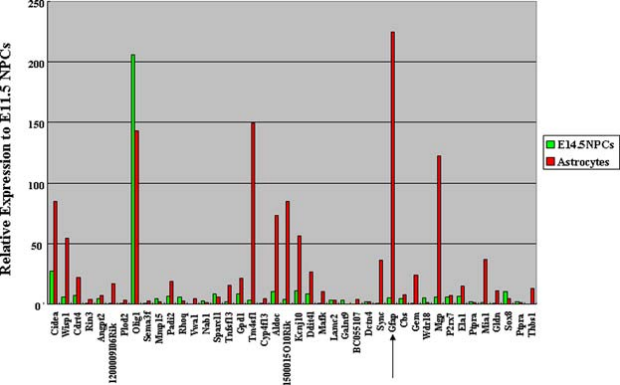
Expression of the genes hypomethylated both in E14.5 NPCs and astrocytes compared to E11.5 NPCs. Among hypomethylated genes, genes which showed upregulation in E14.5 NPCs or astrocytes compared to E11.5 NPCs are shown. Expression is calculated from data obtained by expression microarray analysis and presented as fold change compared to E11.5 NPCs. *Gfap* is indicated by an arrow.

**Figure 6:**
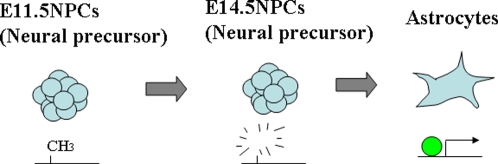
Model proposed for astrocyte-specific expression of the genes demethylated in E14.5 NPCs. Several astrocyte-specific genes are already demethylated in E14.5 NPCs; however, they are induced after diffentiation to astrocytes. This could be explained by astrocyte-specific transcription factors.

Expression changes during the differentiation potential switch of NPCs were analyzed by comparing mid-(E11.5) and late (E14.5)-stage NPCs, and astrocytes by expression microarray analysis (Fig. 4, Table S2). We found that 49% of the probes upregulated in E14.5 NPCs compared to E11.5 NPCs were also upregulated in astroctyes (Fig. 3B). This is smaller than the ratio of hypomethylation where 80% of the probes hypomethylated in E14.5 NPCs were also hypothylated in astrocytes (Fig. 2C). This implies that astrocyte-specific genes, including *Gfap, Aldoc,* and *Kcnj10* (*Kir4.1*), are demethylated at E14.5 NPCs, while they are not expressed until cells differentiate into astrocytes. To examine this hypothesis, we analyzed the expression of genes hypomethylated both in E14.5 NPCs and astrocytes. Among these genes, those that showed upregulation in E14.5 NPCs or astrocytes compared to E11.5 NPCs are shown in Fig. 5. Of these, 64% of the genes, including astrocyte-specific marker *Gfap*, showed more than twice the expression level in astrocytes compared to E14.5 NPCs. Thus, genes already demethylated, such as *Gfap, Aldoc,* and *Kcnj10* (*Kir4.1*), were competent but not highly expressed in E14.5 NPCs, suggesting that their expression appears after differentiation into astrocytes (Fig. 6). Astrocyte-specific genes probably require the expression and/or activation of astrocyte-inducing transcription factors, such as STAT3, to be effectively expressed (Fig. 6).

**Figure 7:**
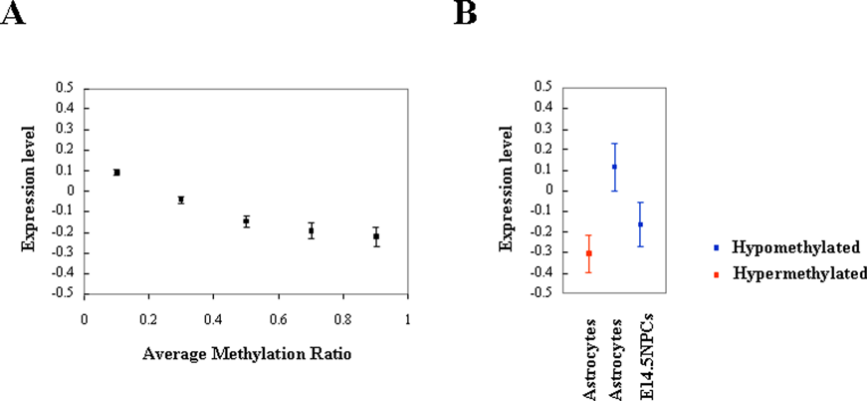
DNA methylation and expression. (A) All probes are grouped by their average methylation ratios and their expression levels are indicated as average±standard error of mean. Expression levels are described as normalized values in log scale. (B) Expression levels of hypermethylated (red) and hypomethylated (blue) genes in astrocytes. Expression levels are indicated as average±standard error of mean.

To analyze the relation between the DNA methylation and the expression of genes, we plotted the methylation ratio calculated by MIAMI and the expression level calculated by expression microarray analysis for all genes examined (Fig. 7A). As expected, the methylation ratio is inversely correlated to the expression level. Next we analyzed the average expression levels of hypermethylated and hypomethylated genes in astrocytes compared to E11.5 NPCs (Fig. 7B). Although there are some exceptions, we found the average expression levels of hypermethylated genes were low and those of hypomethylated genes were high in astrocytes. One of the reasons which could explain the existence of exceptions is the wide distribution of the probes (between −8000 to +2000 bases from the transcription start sites). Interestingly, the expression levels of hypomethylated genes were low in E14.5 NPCs compared to ones in astrocytes. This again indicates activation of expression appears after differentiation into astrocytes.

### STAT and SMAD binding sites in demethylated and upregulated genes

**Figure 8:**
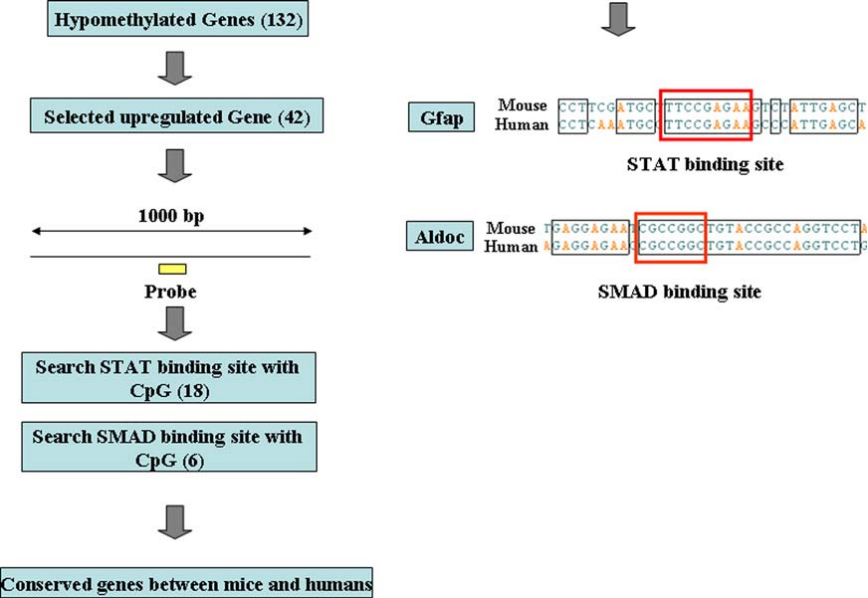
Screening of conserved STAT and SMAD binding sites in demethylated and upregulated genes. Schematic representation of screening of conserved STAT and SMAD binding sites in hypomethylated genes both in E14.5 NPCs and astrocytes. Numbers of genes are indicated in parentheses.

A CpG sequence in a conserved STAT-binding site in the *Gfap* gene promoter is methylated in NPCs midgestation when astrogliogenesis does not normally commence; however, it becomes demethylated in late gestational NPCs that have gained the potential to differentiate into astrocytes [15]. Thus, the *Gfap* gene with the hypomethylated promoter can be expressed upon astrocyte differentiation induced by LIF, which activates the JAK/STAT signaling pathway [13]. Therefore, we searched for conserved STAT-binding sites in the genes hypomethylated both in E14.5 NPCs and astrocytes compared to E11.5 NPCs. To avoid omitting threshold level genes, the threshold used in E14.5 NPCs was extended (D10<0.5). Among these genes, those that showed upregulation in E14.5 NPCs or astrocytes compared to E11.5 NPCs were used for analysis (Fig. 8). Sequences including 500-(basepair) bp upstream and 500-bp downstream of the probes were searched for STAT3 binding sites with the CpG sequence (TTN4-6AA, CG in any N position). Such methylation-sensitive STAT binding sites were found among these 18 genes; however, none were conserved between mice and humans except for *Gfap*, which shows hypomethylation both in E14.5 NPCs and astrocytes (Fig. 1). Although we cannot rule out the possibility that some demethylated STAT binding sites were not detected by the MIAMI method because of its target limitation, these results suggest that not all astrocyte-specific genes are epigenetically controlled only via STAT binding site methylation.

Bone morphogenetic proteins (BMPs) also use STATs to activate the expression of astrocyte marker genes via the association between STATs and the transactivating complex composed of the MBP-activated signaling factors Smad1 and P300/CBP [33], [34]. Therefore, we also searched for conserved SMADs-binding sites in the genes hypomethylated both in E14.5NPCs and astrocytes compared to E11.5 NPCs. We found methylation sensitive SMAD-binding sites in 6 genes; however, only *Aldoc* was conserved between mouse and human (Fig. 8) Bisulfite sequencing analysis of this SMAD sites reveals demethylation in E14.5 NPCs and astrocytes (Fig. 3B).

We could not find methylation-sensitive binding sites for transcription factor except for *Gfap* and *Aldoc*. Effective activation of demethylated genes in E14.5 NPCs probably requires cooperation with other transcription factors, which are activated in astrocyte differentiation.

### Overrepresented genes with methylation changes

Overrepresented and underrepresented categories of genes were searched in hypomethylated and hypermethylated genes. These genes were first classified into lists using PANTHER classification categories (http://www.pantherdb.org/tools/compareToRefListForm.jsp). Each list was then compared to the reference list using the binomial test [35] for each molecular function, biological process, or pathway term in PANTHER. Unexpectedly, there was no significant overrepresentation and underrepresentation in hypomethylated genes in E14.5 NPCs and in astrocytes (P = 0.01). On the other hand, biological process categories such as “mRNA transcription regulation”, “developmental processes”, “mRNA transcription”, “neurogenesis”, “ectoderm development”, “nucleoside, nucleotide and nucleic acids metabolism” were overrepresented among hypermethylated genes in astrocytes (P = 0.01, Table 1). A molecular function category, “transcription factor”, was also overrepresented among hypermethylated genes in astrocytes (P = 0.01, Table 1). It is reasonable that developmental genes are shut off by DNA methylation in terminally differentiated cells. A large part of developmental genes consists of the transcription factor; therefore, it is also reasonable that a category related to transcription factor such as “mRNA transcription regulation” is overrepresented in hypermethylated genes. It is interesting to know that the pathway category, “Notch signaling pathway”, is overrepresented 9-fold more than expected among hypermethylated genes in astrocytes, although it is not statistically significant (P = 0.03). These genes include *Dll1*, *Hes1*, *Hes5*, *Hey2*, and *Ncor2* (Table 2). The Notch signaling pathway has been shown to be important in astrocyte differentiation [36]. Inactivation of developmental and transcriptional factor genes by DNA methylation is one of the important aspects of differentiation. For example, inactivation of transcription factor genes, *Oct3/4* (*Pou5f1*) and *Nanog*, by DNA methylation is important for early development [6], [7]. We also found that transcription factor genes were overrepresented among methylated genes in astrocytes. Regulation by DNA methylation of transcription factor genes is an attractive model to explain development and differentiation. Usually differentiation is caused by transcription factors, which are also regulated by upstream transcription factors. So what is the most upstream regulator? One answer is that the most upstream regulator is epigenetic change.

**Table 1:** Overrepresented category of hypermethylated genes in astrocytes.

Biological Process	Obs/Exp	P-value
Mrna transcription regulation	3.7	1.30E-10
Developmental processes	2.8	1.35E-10
Mrna transcription	2.9	4.32E-08
Neurogenesis	3.5	4.67E-04
Endoderm development	3.2	5.93E-04
Nucleoside, nucleotide and nucleic acid metabolism	1.8	3.19E-03

Molecular Function	Obs/Exp	P-value
Transcription factor	2.9	4.29E-09

**Table 2:** Hypermethylated genes in astrocytes belong to Notch signaling pathway.

Gene Symbol	Gene Name	Probe Pos
Dll1	delta-like 1	1725
Hes1	hairy and enhancer of split 1	−2503
Hes5	hairy and enhancer of split 5	−7260
Hey2	hairy/enhancer-of-split related with YRPW motif 2	−1761
Ncor2	nuclear receptor co-repressor 2	−5790

In conclusion, we suggested in this study that many astrocyte-specific genes are demethylated in common in late-stage NPCs, enabling cells to become competent before astrocyte differentiation. This means DNA demethylation rather than methylation is a critical regulatory event in astrocyte differentiation. We also indicated that group of genes categorized as developmental and transcription factor genes are shut off by DNA methylation in terminally differentiated astrocytes.

## Materials and Methods

### Methylation profiling by MIAMI

The MIAMI method was performed using one microgram of genomic DNA as previously described [16], [17]. The complete experimental procedure can be obtained at http://grc.dept.med.gunma-u.ac.jp/gene/image/MIAMI20Protocol20V4.pdf. The microarray was the same as that used in our previous paper [17]. A brief description of the MIAMI method is as follows.

Changes in methylation were judged by using the difference in methylation-sensitive *Hpa* II cleavage and methylation-insensitive *Msp* I cleavage between samples. To detect differences in methylation-sensitive *Hpa* II cleavage, 0.5 microgram of genomic DNA was digested with 40 units of *Hpa* II overnight in a 100-microliter volume containing 33 mM Tris-acetate (pH7.9), 66 mM KOAc, 10 mM MgOAc_2_, 0.5 mM DTT, and 0.01% BSA. The adaptor was prepared by annealing two oligonucleotides, AGCACTCTCCAGCCTCTCACCGAG and CGCTCGGTGA. After phenol extraction and ethanol precipitation, DNA was ligated to the adaptor with 60 units of E coli DNA Ligase (Takara, Japan). The first PCR was performed using 0.1 microgram of each ligation mix as a template with a primer AGCACTCTCCAGCCTCTCACCGAG using GeneTaq DNA polymerase (Nippon Gene, Japan). The reaction mixture was incubated for 5 min at 72°C and 3min at 94°C and subjected to 5 cycles of amplification consisting of 10 sec denaturation at 94°C, 30 sec annealing at 70°C, 2.5 min extension at 72°C. The final extension was lengthened to 9.5 min. Amplified DNA was digested with *Msp* I by adding 35 units of the enzyme directly to the solution. After 3-hour incubation, the digested DNA was subjected to further amplification by a second PCR. The condition was the same as in the first PCR except for the number of cycles. The optimal sub-saturation cycle number was adopted (usually 10 to 13 cycles). The PCR product was purified with a MiniElute PCR purification Kit (Qiagen, USA). To detect of differences in methylation-insensitive *Msp* I cleavage, 0.5 microgram of genomic DNA was digested with 40 units of *Msp* I overnight and subjected to the same procedure as for the amplification of unmethylated DNA fragments.

Amplified *Hpa* II-cleaved DNA fragments from two samples to be compared were labeled with Cy3 and Cy5, respectively, as described [37], and cohybridized to a microarray. All hybridization procedures except for washing were according to the manual of Agilent Technology (USA). Amplified *Msp* I-cleaved DNA fragments from two samples to be compared were labeled with Cy3 and Cy5, respectively, and cohybridized to another microarray with the same probes.

We judged spots with methylation changes using two models; one in which spots with methylation changes had different values for *Hpa* II and *Msp* I cleavage, and the other in which spots with methylation changes had a large *Hpa* II cleavage difference (more than 5) and a small *Msp* I cleavage difference (less than 2). The reproducibility of the experiment was analyzed with 885 triplicated probes on each microarray and repeated experiments.

### Bisulfite genomic analysis

Bisulfite treatment of genomic DNA was performed using a CpGenome DNA modification kit (INTERGEN). Modified DNA was amplified with the primers described in Table S3. For COBRA analysis, the restriction enzymes are described in Table S3.

### Expression microarray analysis

Expression microarray analysis was performed using the Agilent mouse whole genome array and the procedure provided by Agilent Technologies. A signal ratio of more than 2 with a P-value of less than 0.01 was judged as upregulated. A signal ratio of less than 0.5 with a P-value of less than 0.01 was judged as downregulated.

### Animals and cell preparation

Time-pregnant ICR mice were used to prepare NPCs. The experimental protocols described below were performed according to the animal experimentation guidelines of Nara Institute of Science and Technology. NPCs were prepared from telencephalons of E11.5 and E14.5 mice and cultured as described previously [34]. Briefly, the telencephalons were triturated in Hank's balanced salt solution (HBSS) by mild pipetting with a 1-ml pipet tip (Gilson). Dissociated cells were cultured in N2-supplemented Dulbecco's Modified Eagle's Medium with F12 (GIBCO) containing 10 ng/ml basic FGF (R&D Systems) (N2/DMEM/F12/bFGF) on culture dishes (Nunc) of a chamber slide (Nunc) which had been precoated with poly-L-ornithine (Sigma) and fibronectin (Sigma). For primary astrocyte cultures, the cerebral cortices of postnatal day 1 mice were dissociated using trypsin (GIBCO), and cultured for two weeks in DMEM containing 10% fetal calf serum.

## Supporting Information

Table S1 Table S1 Genome-wide profiling of DNA methylation. Hypomethylated and hypermethylated genes in E14.5 NPCs and astrocytes compared to E11.5 NPCs. Each row indicates each probe. Probes with methylation changes are indicated by color as in Fig. 1A.
(0.06 MB XLS)

Table S2 Table S2 List of upregulated genes compared to E11.5 NPCs. Upregulated genes in E14.5 NPCs and astrocytes compared to E11.5 NPCs.
(0.55 MB XLS)

Table S3 Table S3 List of primers and restriction enzymes used for COBRA and bisulfite sequencing analysis.
(0.02 MB XLS)
